# Supplementary figures and images for: An E3 ubiquitin ligase from Nicotiana benthamiana targets the replicase of Bamboo mosaic virus and restricts its replication
Source: Mol Plant Pathol. 2019 Mar 29;20(5):673–84. doi: 10.1111/mpp.12784 (PMC6637893; doi:10.1111/mpp.12784)

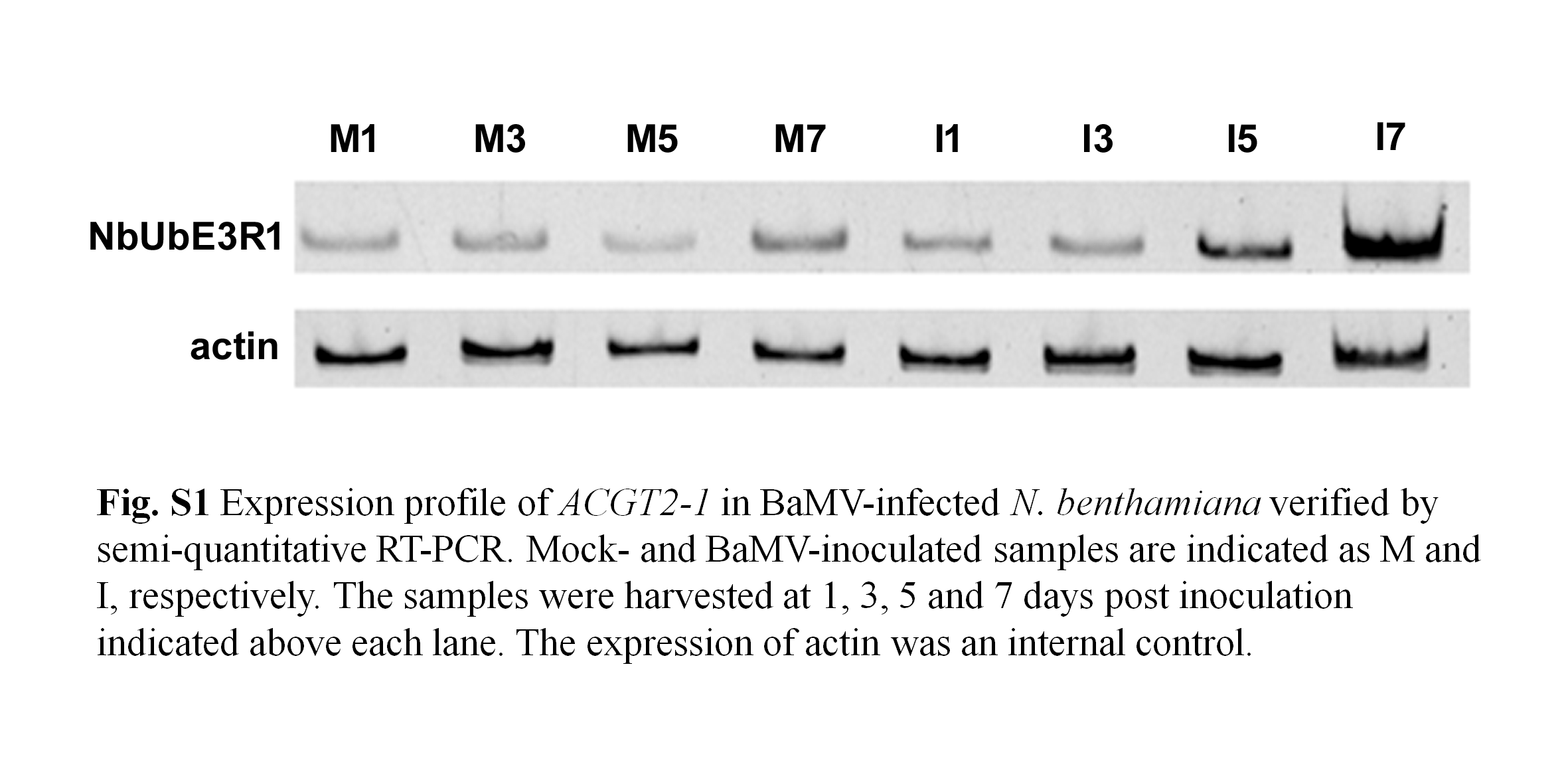

Supplement: Supplementary file 1 — Fig. S1 Expression profile of ACGT2‐1 in BaMV infected N. benthamiana verified by semi quantitative Reverse Transcription Polymerase Chain Reaction (RT PCR). Mock and BaMV inoculated samples are indicated as M and I, respectively. The samples were harvested at 1, 3, 5 and 7 days post inoculation indicated above each lane. The expression of actin was an internal control. [file MPP-20-673-s001.tif]

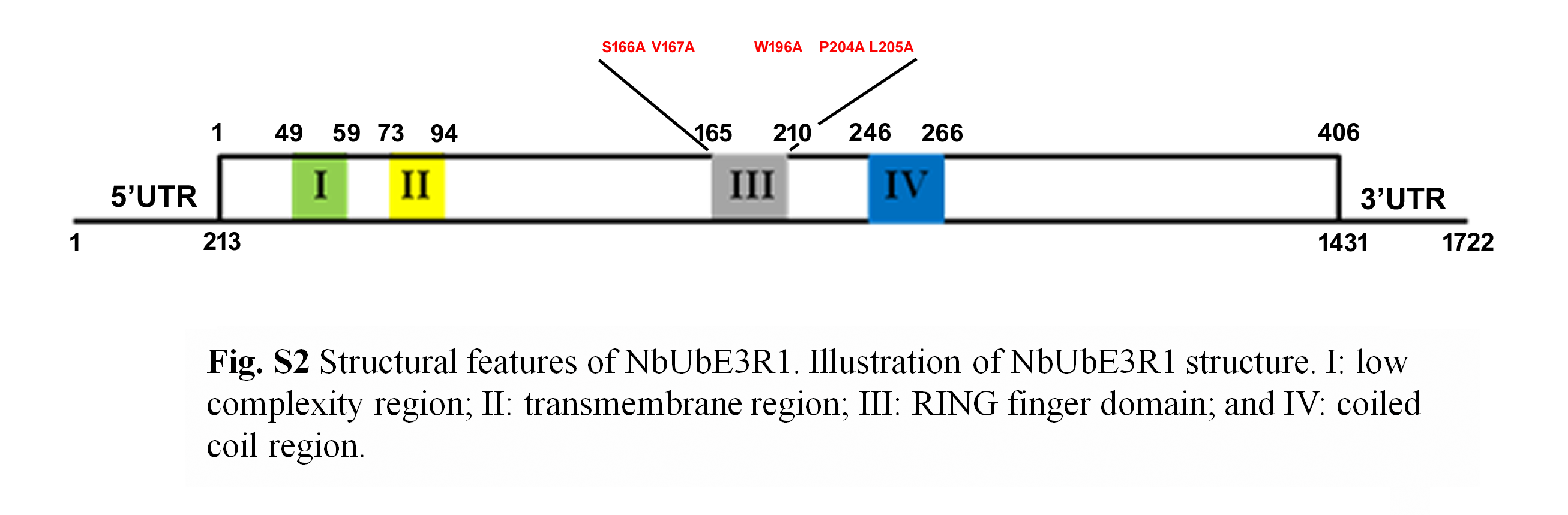

Supplement: Supplementary file 2 — Fig. S2 Structural features of NbUbE3R1. Illustration of NbUbE3R1 structure. I: low complexity region; II: transmembrane region; III: RING finger domain; and IV: coiled coil region. [file MPP-20-673-s002.tif]

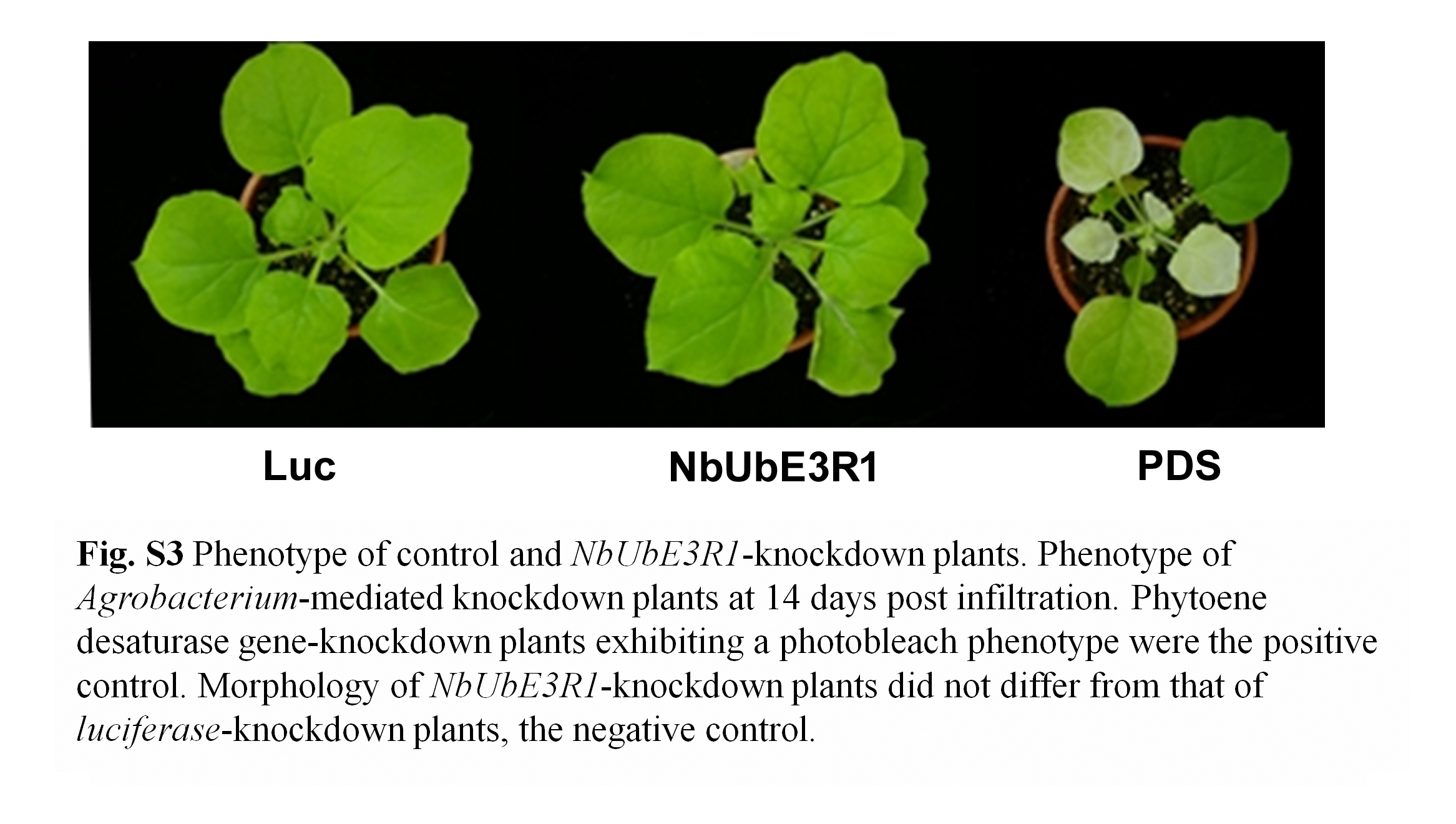

Supplement: Supplementary file 3 — Fig. S3 Phenotype of control and NbUbE3R1 knockdown plants. Phenotype of Agrobacterium mediated knockdown plants at 14 days post infiltration. Phytoene desaturase gene knockdown plants exhibiting a photobleach phenotype were the positive control. Morphology of NbUbE3R1 knockdown plants did not differ from that of Luciferase knockdown plants, the negative control. [file MPP-20-673-s003.tif]
